# Global Trends in the Affordability of Sugar-Sweetened Beverages, 1990–2016

**DOI:** 10.5888/pcd14.160406

**Published:** 2017-05-04

**Authors:** Evan Blecher, Alex C. Liber, Jeffrey M. Drope, Binh Nguyen, Michal Stoklosa

**Affiliations:** 1 Institute for Health Research and Policy, University of Illinois at Chicago, Chicago, Illinois; 2 Economic and Health Policy Research, American Cancer Society, Atlanta, Georgia; 3 Independent Consultant, Palo Alto, California

## Abstract

**Introduction:**

The objective of this study was to quantify changes in the affordability of sugar-sweetened beverages, a product implicated as a contributor to rising rates of obesity worldwide, as a function of product price and personal income.

**Methods:**

We used international survey data in a retrospective analysis of 40 high-income and 42 low-income and middle-income countries from 1990 to 2016. Prices of sugar-sweetened beverages were from the Economist Intelligence Unit’s World Cost of Living Survey. Income and inflation data were from the International Monetary Fund’s World Economic Outlook Database. The measure of affordability was the average annual percentage change in the relative-income price of sugar-sweetened beverages, which is the annual rate of change in the proportion of per capita gross domestic product needed to purchase 100 L of Coca-Cola in each country in each year of the study.

**Results:**

In 79 of 82 countries, the proportion of income needed to purchase sugar-sweetened beverages declined on average (using annual measures) during the study period. This pattern, described as an increase in the affordability of sugar-sweetened beverages, indicated that sugar-sweetened beverages became more affordable more rapidly in low-income and middle-income countries than in high-income countries, a fact largely attributable to the higher rate of income growth in those countries than to a decline in the real price of sugar-sweetened beverages.

**Conclusion:**

Without deliberate policy action to raise prices, sugar-sweetened beverages are likely to become more affordable and more widely consumed around the world.

## Introduction

In recent years the consumption of sugar-sweetened beverages has increased in both high-income countries (HICs) and low-income and middle-income countries (LMICs) (1–3). Because sugar-sweetened beverage consumption is linked to obesity and overweight — risk factors for many noncommunicable diseases, including cardiovascular diseases, many cancers, and type 2 diabetes — this trend has generated concern in the public health community (4–6). Obesity and overweight can be modifiable risk factors, and decreases in consumption of sugar-sweetened beverages tend to reduce their prevalence and therefore the prevalence of disease (7,8).

Overweight and obesity are reaching record-high levels. From 2000 to 2013, the global prevalence of overweight and obesity increased by one-quarter among adults and by nearly half among children; by 2013, more than 2 billion people were overweight, and of these, more than 674 million were obese (9). In 2010, overweight and obesity were estimated to cause 3.4 million deaths worldwide (10), a number that is likely to increase in line with rising overweight and obesity rates. The annual health care costs attributable to obesity and overweight are more than $600 billion (11).

As with the consumption of most other consumer products, consumption of sugar-sweetened beverage tends to rise when prices decline (12). Similarly, sugar-sweetened beverage consumption also tends to grow as consumer income grows (2,13). This increased consumption can negatively affect health (12) with potentially heightened effects in discrete groups such as young people or certain racial/ethnic groups (2,14,15). Scholars have examined the broader implications of tax and price increases on consumption of sugar-sweetened beverages, including implications for discrete groups such as lower-income households (13,16). An increasing number of experts, including a recent panel at the World Health Organization, overtly promote interventions that will affect price, most commonly excise taxation (17–19).

The strategies of the soft drink industry also likely influence sugar-sweetened beverage consumption. In particular, the industry undermines people’s ability to resist overconsuming sugar-sweetened beverages by lowering the unit price as product size increases (20). The industry also spends hundreds of millions every year globally to advertise sugar-sweetened beverages, especially to children and adolescents (21). Of the 2 largest sugar-sweetened beverage manufacturers, the Coca-Cola Company spent the 8th most and PepsiCo spent the 10th most among all corporations to advertise their products globally in 2013 (22). To facilitate these marketing strategies, Coca-Cola and many similar firms have created extensive and well-organized global distribution networks guaranteeing the ubiquity of their products (23).

To understand and predict consumers’ decisions on sugar-sweetened beverage purchases, many studies have focused on trends in food prices (24,25). Price alone, however, is an imperfect predictor of consumers’ purchasing behaviors. With many LMICs experiencing high rates of economic growth, it is necessary to focus on the effect of personal income on consumption as well. Addressing the shortcoming of research that has historically conceptualized price and income in isolation, recent public health research has begun to focus on affordability, which measures changes in prices and income simultaneously. Measures of affordability are typically expressed as the percentage of a worker’s income (26) or duration of work time required to buy a defined amount of product (27) or both (28,29).

The measurement of affordability of other products, such as cigarettes and alcohol — both major risk factors for noncommunicable disease — is common (29–31). Alcohol has become more affordable over time, and increases in affordability have been driven largely by increases in incomes (32). Research on cigarette affordability demonstrates divergent results between HICs and LMICs. Cigarettes are less affordable in HICs where there are concerted efforts to raise prices through increased excise taxes; however, they are more affordable in most LMICs where rapid increases in incomes outpace any increases in taxes and prices (30).

To date, no research has measured systematically the affordability of sugar-sweetened beverages or trends thereof, although some researchers have considered the concept of affordability of sugar-sweetened beverages or the relative prices of other foods and sugar-sweetened beverages, a related but less powerful conceptualization of affordability (1,12). On the other hand, the soft drink industry is already using the affordability concept, as Coca-Cola’s marketing strategy has explicitly identified increasing affordability of sugar-sweetened beverages as a key growth strategy (23).

We argue that affordability is an indispensable measure to understand and predict consumers’ decisions on sugar-sweetened beverage purchases. Affordability should definitively influence the trajectory of the global overweight and obesity epidemic because it takes into account the simultaneous effect of price and income on consumers’ purchasing behaviors. Our study applies existing techniques used mostly to evaluate the affordability of cigarettes and alcohol to measure the affordability of sugar-sweetened beverages. We compared the most recent estimates of affordability (in 2016) among countries, trends in affordability from 1990 through 2016, and the decomposition of price and income effects on changes in affordability.

## Methods

The main reason to examine both changes in prices and affordability is that the magnitude of the changes in prices and affordability is most likely to affect behavior over time. Accordingly, we measured trends by using the average annual percentage change, which was estimated by using the constant growth regression technique (33). To consider changes in prices over time, nominal prices were converted to real prices to take into account inflation (in constant 2010 prices). We used the relative-income price method to measure the affordability of sugar-sweetened beverages. This method was developed to measure trends in affordability of cigarettes (29). The method uses a broad measure of income (per capita gross domestic product [GDP]), which is appropriate for research on LMICs because it is a comprehensive measure of economic activity, for example, by including crucial components of the economy such as the value of public goods and services as well as transfer payments. Although not perfect (for example, in some HICs, some evidence suggests that wages and/or household incomes are not keeping up with overall growth for some discrete groups [34,35]), this method also permits the calculation of affordability for the largest group of countries and the longest possible annual time series. Narrower measures of income, such as wages, are not as widely and frequently available as GDP and thus would limit the number of countries, particularly the number of LMICs, and the length and frequency of the time series (30). Narrow measures of income may be preferable for individual country studies or for groups of countries where such data are readily available.

The relative-income price for cigarettes measures the percentage of per capita GDP needed to purchase 100 packs of cigarettes per year. We adapted this measure to estimate the percentage of per capita GDP to purchase 100 L of sugar-sweetened beverages. A higher relative-income price means that sugar-sweetened beverages are less affordable than in a country or time period with a lower relative-income price, and an increase in relative-income price means that sugar-sweetened beverages have become less affordable. The relative-income price is a relative concept and has no absolute meaning; that is, a sugar-sweetened beverage cannot be affordable or unaffordable, but rather can be more or less affordable than in another country or time period.

We used Coca-Cola as a proxy for all sugar-sweetened beverages because it is the most globally recognizable sugar-sweetened beverage brand and largely homogeneous. The greater availability of data for this product also permits us to include many more countries and a longer time series. Coca-Cola is the largest carbonated soft drink brand in the world, comprising 25.8% of the global market in 2014, more than double its closest competitor. Including Coca-Cola derivatives (eg, Diet Coke), the brand accounts for 32.8% of the global market for carbonated soft drinks (36). Furthermore, some research suggests that despite complexity in pricing among sugar-sweetened beverage firms, prices for like-products commonly converge (37).

Price data were drawn from the Economist Intelligence Unit’s World Cost of Living Survey (38). This survey annually collects prices on many consumer goods. Sugar-sweetened beverages are represented by the price of 1 L of Coca-Cola in 2 retail environments (a supermarket and a mid-priced store). By convention, we used the lowest price of the two (in some countries and/or years only one environment is surveyed) (29,30). The survey collects prices by city. In many countries only a single city is surveyed, and we used data for that city. However, in some countries, multiples cities are surveyed, and we used the average price. When cities were added at a later stage or during inconsistent time periods, by convention, we did not include the additional cities (29,30).

Income data were collected from the International Monetary Fund’s World Economic Outlook Database (39) in the local currency to calculate the relative-income price. During periods of hyperinflation, US dollar values were used instead. Real prices for those countries were estimated in US dollars as well. Countries were categorized as either an HIC or an LMIC according to the World Bank’s most recent classification. The sample consisted of 82 countries: 42 LMICs and 40 HICs.

The analysis was conducted in 3 parts. We first considered the differences among countries in 2016 for prices in US dollars and the relative-income price. Second, we considered the trends in real prices and the relative-income price from 1990 through 2016. Third, because changes in affordability can be driven by declines in prices or increases in incomes or a combination of the two, it is useful to decompose the change in affordability into the changes in prices and incomes. To illustrate this, we subtracted the average annual percentage change of price from the average annual percentage change of relative-income price, thereby estimating the average annual percentage change of income and then stacking the price and income components. Because an increase in price increases the relative-income price but an increase in income decreases the relative-income price, we inverted the income series so that the stacked components combined to measure the change in affordability. We then ordered the series by the change in affordability.

## Results

### Price and affordability

**Figure 1:**
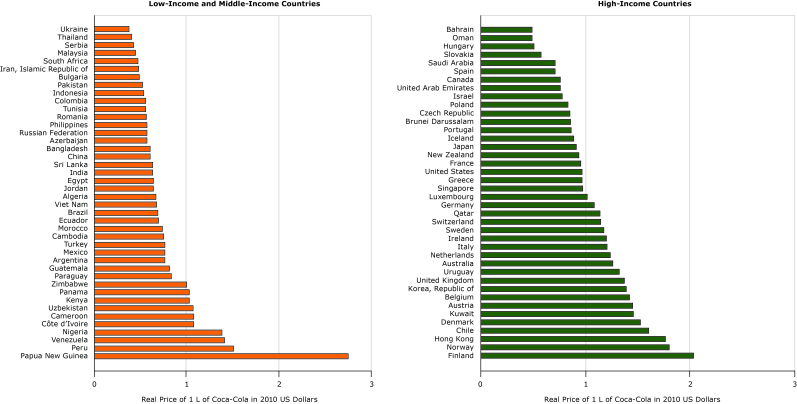
Real price of 1 L of Coca-Cola in 40 high-income and 42 low-income and middle-income countries, in 2010 US dollars, 2016.

The mean (standard deviation) price of 1 L of Coca-Cola in 2016 was $0.73 ($0.26) among all countries surveyed (Table 1); the price varied considerably around the world, ranging from $0.38 per liter in Ukraine to $2.74 per liter in Papua New Guinea (Figure 1). Both the lowest and highest prices were in LMICs. The mean and median prices for sugar-sweetened beverages were higher in HICs than in LMICs, although the differences were not large (Table 1). We found substantial variation in both the LMIC and HIC groups, although the within-group variation was similar.

**Table 1:** Summary Statistics for Prices and Relative-Income Prices of Sugar-Sweetened Beverages in 2016^a^

Statistic	No. of Countries	Mean Price (SD)	Median Price (IQR)	Coefficient of Variation^b^
**Price, $**				
Low-income and middle-income countries	42	0.66 (0.21)	0.60 (0.03)	0.32
High-income countries	40	1.03 (0.24)	0.96 (0.30)	0.24
All countries	82	0.73 (0.26)	0.64 (0.16)	0.36
**Relative-income price^c^, %**				
Low-income and middle-income countries	42	2.48 (1.88)	1.56 (3.30)	0.76
High-income countries	40	0.33 (0.19)	0.28 (0.18)	0.59
All countries	82	2.08 (1.89)	0.95 (3.30)	0.91

Abbreviations: IQR, interquartile range, SD, standard deviation.

a Estimates are weighted by total country population in 2016. Prices are in 2010 US dollars. Data sources: Economist Intelligence Unit’s World Cost of Living Survey (38) and International Monetary Fund’s World Economic Outlook Database (39).

b Coefficient of variation is the ratio of the standard deviation to the mean, which facilitates the comparison of variation between 2 measures. A comparison of coefficients of variation in this table demonstrates that between countries, relative-income price varies more than real price.

c Percentage of per capita gross national product to purchase 100 L of sugar-sweetened beverages.

**Figure 2:**
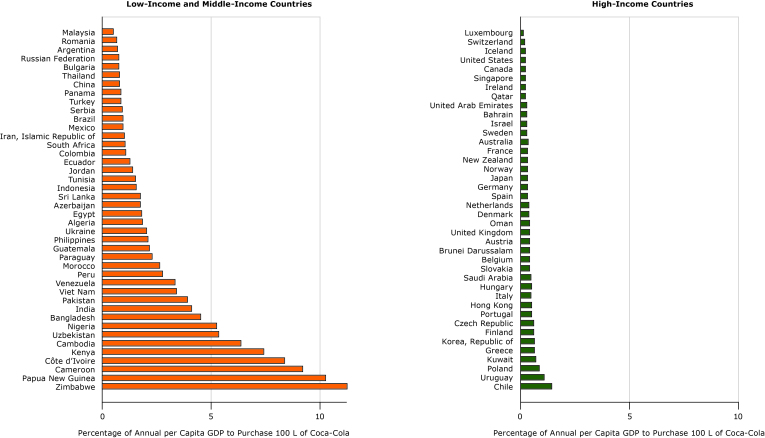
Relative-income price of 100 L of Coca-Cola in 40 high-income and 42 low-income and middle-income countries, 2016. Units are percentage of annual per capita gross domestic product (GDP) to purchase 100 L of Coca-Cola.

We found a different result in the affordability of sugar-sweetened beverages (Figure 2). The relative-income price of sugar-sweetened beverages varied, ranging from 0.11% of annual per capita GDP to purchase 100 L in Luxembourg to 11.24% in Zimbabwe. The lowest relative-income prices were found in HICs and the highest relative-income prices were found in LMICs. Sugar-sweetened beverages were substantially more affordable in almost all HICs than in LMICs.

### Changes in prices and affordability between 1990 and 2016

**Figure 3:**
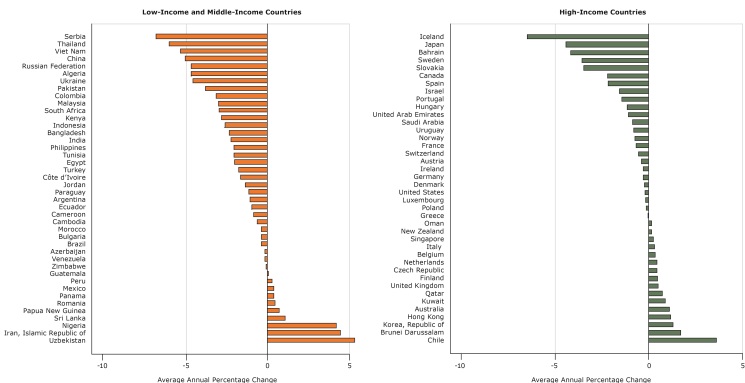
Average annual percentage change in real prices of sugar-sweetened beverages in 40 high-income and 42 low-income and middle-income countries from 1990 to 2016.

The real prices of sugar-sweetened beverages increased in 26 countries and declined in 56 countries on average annually between 1990 and 2016 (Figure 3). In HICs, the real price increased in 16 countries and decreased in 24 countries; in LMICs, real prices increased in 10 countries, and decreased in 32 countries. At the average and the median, real prices and relative-income prices of sugar-sweetened beverages declined in both LMICs and HICs (Table 2); however, we found wide within-group variation.

**Table 2:** Summary Statistics of Average Annual Percentage Change for Real Income, Real Prices, Relative-Income Price, and Consumption of Sugar-Sweetened Beverages from 1990 to 2016^a^

Statistic	No. of Countries	Mean (SD), %	Median (IQR), %	Coefficient of Variation^b^
**Real income**				
Low-income and middle-income countries	42	6.07 (3.38)	4.97 (7.43)	0.56
High-income countries	40	1.27 (1.66)	1.06 (1.27)	1.31
All countries	82	5.19 (3.63)	4.97 (5.29)	0.70
**Real prices**				
Low-income and middle-income countries	42	−2.69 (2.45)	−2.36 (2.81)	−0.91
High-income countries	40	−0.69 (1.66)	−0.20 (1.00)	−2.42
All countries	82	−2.32 (2.44)	−2.27 (4.31)	−1.05
**Relative-income price^c^**				
Low-income and middle-income countries	42	−8.76 (5.00)	−7.24 (10.39)	−0.57
High-income countries	40	−1.96 (1.52)	−1.26 (1.83)	−0.78
All countries	82	−7.52 (5.25)	−7.06 (9.01)	−0.70
**Consumption**				
Low-income and middle-income countries	35	4.39 (2.16)	5.64 (2.84)	0.49
High-income countries	33	−0.35 (2.19)	−0.77 (3.40)	−6.19
All countries	68	3.50 (2.85)	5.23 (4.29)	0.82

Abbreviations: IQR, interquartile range, SD, standard deviation.

a Estimates are weighted by total country population in 2016. Consumption estimates as of 2015; consumption estimates were available for 68 countries only. Data sources: Euromonitor International (36), Economist Intelligence Unit’s World Cost of Living Survey (38), and International Monetary Fund’s World Economic Outlook Database (39).

b Coefficient of variation is the ratio of the standard deviation to the mean, which facilitates the comparison of variation between 2 measures. A comparison of coefficients of variation in this table demonstrates that between countries, relative-income price varies more than real price.

c Percentage of per capita gross national product to purchase 100 L of sugar-sweetened beverages.

**Figure 4:**
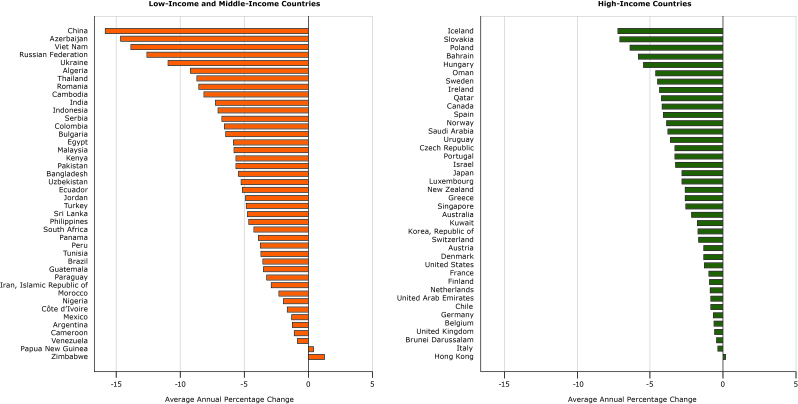
Average annual percentage change in relative-income price of sugar-sweetened beverages in 40 high-income and 42 low-income and middle-income countries from 1990 to 2016.

Sugar-sweetened beverages became more affordable on average annually between 1990 and 2016 in most countries; they became less affordable in only 3 countries or territories (Hong Kong, Papua New Guinea, and Zimbabwe) (Figure 4). Although the average annual percentage change in affordability varied within groups (LMICs and HICs), sugar-sweetened beverages became more affordable more rapidly in LMICs (by an average of 8.76% per year) than in HICs (an average of 1.96% per year) (Table 2).

**Figure 5:**
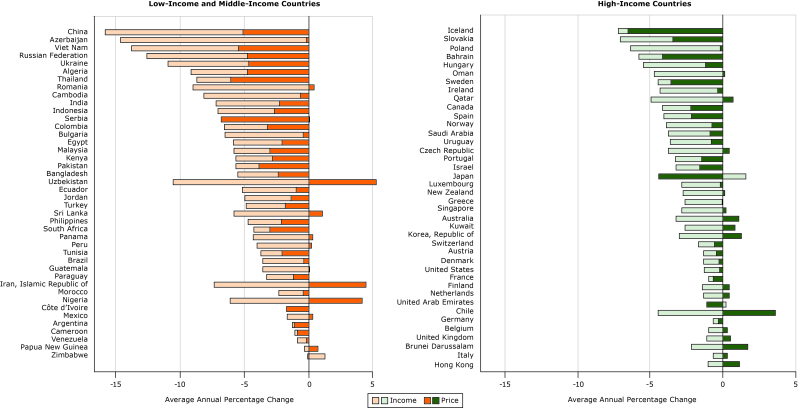
Decomposition of effects of income and price of sugar-sweetened beverages in 40 high-income and 42 low-income and middle-income countries from 1990 to 2016. Units are average annual percentage change. **Table:** 

The country with the largest annual average increase in affordability (or negative average annual percentage change of relative-income price) between 1990 and 2016 was China (Figure 5), where prices declined and incomes increased (income was inverted so it appears negative, but is in fact positive to the same magnitude). Conversely, prices increased on average annually in the Islamic Republic of Iran (hence a positive value in the figure) and so did income (again plotted as a negative value with respect to affordability), but because the increase in income was greater than the increase in price, affordability increased and relative-income price declined.

Country	Price, US Dollars	Income, US Dollars
**Low-Income and Middle-Income Countries (n = 42)**		
China	−5.08	−10.77
Azerbaijan	−0.17	−14.46
Viet Nam	−5.38	−8.46
Russian Federation	−4.71	−7.86
Ukraine	−4.61	−6.31
Algeria	−4.71	−4.48
Thailand	−6.04	−2.63
Romania	0.47	−9.03
Cambodia	−0.61	−7.54
India	−2.27	−4.97
Indonesia	−2.66	−4.41
Serbia	−6.84	0.09
Colombia	−3.20	−3.34
Bulgaria	−0.40	−6.06
Egypt	−2.04	−3.82
Malaysia	−3.02	−2.77
Kenya	−2.84	−2.79
Pakistan	−3.84	−1.79
Bangladesh	−2.36	−3.10
Uzbekistan	5.31	−10.55
Ecuador	−0.97	−4.16
Jordan	−1.36	−3.55
Turkey	−1.81	−3.02
Sri Lanka	1.10	−5.82
Philippines	−2.08	−2.57
South Africa	−3.02	−1.21
Panama	0.38	−4.27
Peru	0.26	−3.98
Tunisia	−2.06	−1.63
Brazil	−0.38	−3.19
Guatemala	0.06	−3.55
Paraguay	−1.16	−2.08
Iran, Islamic Republic of	4.48	−7.36
Morocco	−0.40	−1.91
Nigeria	4.20	−6.12
Cote d’Ivoire	−1.69	0.04
Mexico	0.38	−1.66
Argentina	−1.08	−0.15
Cameroon	−0.85	−0.22
Venezuela	−0.16	−0.69
Papua New Guinea	0.75	−0.35
Zimbabwe	−0.08	1.34
**High-Income Countries (n = 40)**		
Iceland	−6.50	−0.68
Slovakia	−3.44	−3.62
Poland	−0.15	−6.20
Bahrain	−4.16	−1.62
Hungary	−1.17	−4.27
Oman	0.13	−4.72
Sweden	−3.55	−0.92
Ireland	−0.32	−4.00
Qatar	0.73	−4.92
Canada	−2.19	−1.95
Spain	−2.14	−1.92
Norway	−0.74	−3.13
Saudi Arabia	−0.85	−2.89
Uruguay	−0.78	−2.81
Czech Republic	0.46	−3.75
Portugal	−1.45	−1.83
Israel	−1.57	−1.65
Japan	−4.39	1.59
Luxemburg	−0.16	−2.63
New Zealand	0.14	−2.73
Greece	−0.03	−2.55
Singapore	0.24	−2.80
Australia	1.10	−3.23
Kuwait	0.84	−2.57
Korea, Republic of	1.28	−2.98
Switzerland	−0.56	−1.08
Austria	−0.42	−0.89
Denmark	−0.24	−1.07
United States	−0.20	−1.06
France	−0.66	−0.31
Finland	0.47	−1.39
Netherlands	0.44	−1.31
United Arab Emirates	−1.09	0.25
Chile	3.61	−4.44
Germany	−0.32	−0.34
Belgium	0.32	−0.94
United Kingdom	0.52	−1.08
Brunei Darussalam	1.72	−2.13
Italy	0.31	−0.65
Hong Kong	1.17	−1.00

Affordability increased in most countries because of a combination of increases in income and decreases in price (Figure 5). Price increases counteracted income increases in only 10 of 42 LMICs and 16 of 40 HICs; however, in all these countries, the increases in prices were not large enough to make sugar-sweetened beverages less affordable. Prices decreased and incomes increased simultaneously in most countries (29 of 42 LMICs and 22 of 40 HICs), increasing affordability. In 9 LMICs and 6 HICs, declining prices contributed more than increasing incomes to the increasing affordability, while in 29 LMICs and 31 HICs increases in incomes contributed more to increases in affordability. Thus, the increases in affordability over time tended to be driven more by developments in income rather than developments in prices.

## Discussion

Sugar-sweetened beverages became more affordable during the 27-year period from 1990 to 2016 in 79 of 82 countries in our data sample. If income had not grown, the changes in prices would have informed us about trends in consumption, but as our research shows, many countries, particularly LMICs, experienced high rates of economic growth in the last 2 decades. The average annual per capita GDP growth in LMICs was 6.07%, while the average annual per capita GDP growth in HICs was 1.27%. As such, growth in income may outweigh increases in prices, and hence the need to consider the changes in affordability over time. From an economic development perspective, policy makers want to engender income growth, so this dynamic reinforces the concept that governments will need to pursue policy interventions on the price side — not the income side — to affect affordability, for example, as many have done with cigarettes through effective excise taxation.

 Although the increase in affordability is partly due to economic progress that resulted from rapid global economic development, it is also attributable to a lack of action taken by policy makers to affect the price of sugar-sweetened beverages, for example, through targeted excise tax increases. This inaction is arguably predictable because the idea of taxing sugar-sweetened beverages arose in the health economics literature late during the study period and has not progressed to become a widely adopted policy option (20) even at the time of writing in early 2017. For example, the implementation of such taxes in France in 2012, Hungary in 2011, and Mexico in 2014 may have occurred too late in the study period to make a meaningful impact on our results.

We argue, and the scientific literature strongly suggests, that this environment of increasingly affordable sugar-sweetened beverages will inevitably drive increased consumption of such products and will certainly hamper global efforts to address the overweight and obesity epidemic. Although sugar-sweetened beverages are not the sole consumer product contributing to the epidemic, their role as a major contributor is difficult to dispute. Just as in the fight against tobacco use, all options for effective intervention deserve consideration. Our research identifies a measureable problem that is contributing to the obesity epidemic and suggests that using affordability as an indicator of policy progress or failure can point policy makers in the right direction for creating price-focused policy interventions. Finally, without deliberate policy action to raise prices, sugar-sweetened beverages are likely to continue to become more affordable and thus more widely consumed around the world.

Policy options to affect the prices of sugar-sweetened beverages might include imposing, restructuring, or increasing excise taxes; mandating a minimum unit price; or preventing discounted products. Some countries, including Mexico, France, and Hungary, have already begun using excise taxes to increase the price of sugar-sweetened beverages (40,41), and preliminary findings from Mexico indicate that their sugar-sweetened beverage excise tax has driven down consumption of sugar-sweetened beverages (40,42).

Policy makers are now using affordability as a measure of progress in cigarette tax policy to significant positive effect (30). The most fundamental barrier to being able to use the tools of affordability to address sugar-sweetened beverage consumption is the willingness to identify sugar-sweetened beverage consumption as a threat to public health that necessitates policy remedy. Such a policy remedy can be taken to intentionally raise the price of sugar-sweetened beverages to keep pace with and eventually outstrip gains in income to make the products less affordable and in turn, consumed less over time.

Although this research demonstrates unequivocally that sugar-sweetened beverages have become more affordable in nearly every corner of the globe, skeptics may suggest that if income is the main driver of affordability in this context then most goods are becoming more affordable. Moreover, this issue could be particularly relevant to substitutes or near-substitutes, some of which could be healthier alternatives to sugar-sweetened beverages. Accordingly, we compared trends for bottled water and sugar-sweetened beverages to provide a control to our examination of sugar-sweetened beverage prices and found that bottled water is typically more expensive and less affordable than sugar-sweetened beverages (Appendix).

Our analysis was somewhat constrained by the available data. We had data for 82 countries, which means that dozens of countries, mainly LMICs, were not included in our analysis, although we had no a priori reason to assume that these countries were different from the LMICs in the sample. Consistent price data were available from 1990 only. The price data were for Coca-Cola only; this product may be priced slightly differently than other brands. We used GDP per capita to calculate relative-income price, a measure with both strengths and weaknesses, discussed above.

The increasing worldwide affordability of sugar-sweetened beverages is likely having an effect on the global overweight and obesity epidemic. More affordable products lead to greater consumption, and increased consumption of sugar-sweetened beverages contributes to overweight and obesity. The change in affordability is a function of declining prices in many countries and increasing incomes. Societies typically want to engender growth, so the logical intervention is for governments to affect prices through excise taxation, as they have done with other unhealthful products such as cigarettes.

## Appendix Comparison of Bottled Water and Sugar-Sweetened Beverage Prices

We compared trends for bottled water and sugar-sweetened beverage prices to provide a control to our examination of sugar-sweetened beverage prices. Although many substitutes exist for sugar-sweetened beverages, water is the one that is arguably least likely to be taxed, and other substitutes, such as 100% fruit juice, also concern many public health specialists because of their high sugar content (43).

Using the same Economist Intelligence Unit data source for bottled water that we used for sugar-sweetened beverages, we replicated many of our analyses. Sugar-sweetened beverage prices in 2016 were lower than bottled water prices in both HICs and LMICs. Average and median bottled water prices were higher in LMICs than in HICs, although the differences were not large. The real price of bottled water increased in 35 countries and declined in 47 countries on average annually from 1990 to 2016. Although bottled water prices declined in most countries, it became less affordable in only 13 countries (6 HICs and 7 LMICs). Bottled water became more affordable by 7.68% per year on average in LMICs, while in HICs, bottled water became more affordable by 1.91% per year.

Although bottled water prices declined in some countries, bottled water is still typically more expensive than sugar-sweetened beverages. Our comparison was limited by the fact that bottled water is not a perfect substitute for sugar-sweetened beverages. The reasons that people drink these beverages are not the same; for example, because bottled water is often a necessity in some regions with substandard public water, and because sugar-sweetened beverages arguably offer a different sensory experience attributable to their flavor and carbonation (44,45).

**Table A1:** Summary Statistics for Price and Relative-Income Price of Sugar-Sweetened Beverages and Bottled Water in 2016^a^

Statistic	No. of Countries	Sugar-Sweetened Beverages			Bottled Water		
		Mean (SD)	Median (IQR)	Coefficient of Variation^b^	Mean (SD)	Median (IQR)	Coefficient of Variation^b^
**Price,** $							
Low-income and middle-income countries	42	0.66 (0.21)	0.60 (0.03)	0.32	1.64 (1.20)	1.92 (2.34)	0.73
High-income countries	40	1.03 (0.24)	0.96 (0.30)	0.24	1.48 (0.53)	1.72 (0.64)	0.36
All countries	82	0.73 (0.26)	0.64 (0.16)	0.36	1.61 (1.10)	1.72 (2.34)	0.68
**Relative-income price^c^, %**							
Low-income and middle-income countries	42	2.48 (1.88)	1.56 (3.30)	0.76	3.86 (2.88)	3.48 (2.24)	0.75
High-income countries	40	0.33 (0.19)	0.28 (0.18)	0.59	0.48 (0.34)	0.33 (0.64)	0.71
All countries	82	2.08 (1.89)	0.95 (3.30)	0.91	3.24 (2.91)	2.54 (1.60)	0.90

Abbreviations: IQR, interquartile range, SD, standard deviation.

^a^ Estimates are weighted by total country population in 2016. Data sources: Economist Intelligence Unit’s World Cost of Living Survey (38) and International Monetary Fund’s World Economic Outlook Database (39).

^b^ Coefficient of variation is the ratio of the standard deviation to the mean, which facilitates the comparison of variation between 2 measures. A comparison of coefficients of variation in this table demonstrates that between countries, relative-income price varies more than real price.

^c^ Percentage of per capita gross national product to purchase 100 L of sugar-sweetened beverages or bottled water.

**Table A2:** Summary Statistics of Average Annual Percentage Change for Real Prices and Relative-Income Price for Sugar-Sweetened Beverages from 1990 to 2016^a^

Statistic	No. of Countries	Sugar-Sweetened Beverages			Bottled Water		
		Mean (SD)	Median (IQR)	Coefficient of Variation^b^	Mean (SD)	Median (IQR)	Coefficient of Variation^b^
**Real income**							
Low-income and middle-income countries	42	6.07 (3.38)	4.97 (7.43)	0.56	6.07 (3.38)	4.97 (7.43)	0.56
High-income countries	40	1.27 (1.66)	1.06 (1.27)	1.31	1.27 (1.66)	1.06 (1.27)	1.31
All countries	82	5.19 (3.63)	4.97 (5.29)	0.70	5.19 (3.63)	4.97 (5.29)	0.70
**Real prices**							
Low-income and middle-income countries	42	−2.69 (2.45)	−2.36 (2.81)	−0.91	−1.56 (3.48)	−2.47 (1.42)	−2.23
High-income countries	40	−0.69 (1.66)	−0.20 (1.00)	−2.42	−0.60 (2.43)	−0.52 (0.92)	−4.06
All countries	82	−2.32 (2.44)	−2.27 (4.31)	−1.05	−1.38 (3.32)	−2.47 (2.36)	−2.40
**Relative-income price^c^**							
Low-income and middle-income countries	42	−8.76 (5.00)	−7.24 (10.39)	−0.57	−7.68 (5.26)	−7.73 (7.70)	−0.69
High-income countries	40	−1.96 (1.52)	−1.26 (1.83)	−0.78	−1.91 (1.64)	−1.59 (0.83)	−0.86
All countries	82	−7.52 (5.25)	−7.06 (9.01)	−0.70	−6.62 (5.28)	−7.73 (9.89)	−0.80
**Consumption**							
Low-income and middle-income countries	35	4.39 (2.16)	5.64 (2.84)	0.49	12.30 (5.25)	12.41 (10.80)	0.43
High-income countries	33	−0.35 (2.19)	−0.77 (3.40)	−6.19	4.46 (2.70)	5.61 (3.21)	0.60
All countries	68	3.50 (2.85)	5.23 (4.29)	0.82	10.82 (5.75)	12.41 (11.86)	0.53

Abbreviations: IQR, interquartile range, SD, standard deviation.

^a^ Estimates are weighted by total country population in 2016. Consumption estimates as of 2015; consumption estimates were available for 68 countries only. Data sources: Euromonitor International (36), Economist Intelligence Unit’s World Cost of Living Survey (38) and International Monetary Fund’s World Economic Outlook Database (39).

^b^ Coefficient of variation is the ratio of the standard deviation to the mean, which facilitates the comparison of variation between 2 measures. A comparison of coefficients of variation in this table demonstrates that between countries, relative-income price varies more than real price.

^c^ Percentage of per capita gross national product to purchase 100 L of sugar-sweetened beverages or bottled water.
